# Nucleosome proteostasis and histone turnover

**DOI:** 10.3389/fmolb.2022.990006

**Published:** 2022-09-30

**Authors:** Adrian Arrieta, Thomas M. Vondriska

**Affiliations:** ^1^ Departments of Anesthesiology and Perioperative Medicine, David Geffen School of Medicine, University of California, Los Angeles, Los Angeles, CA, United States; ^2^ Departments of Medicine/Cardiology, David Geffen School of Medicine, University of California, Los Angeles, Los Angeles, CA, United States; ^3^ Departments of Physiology, David Geffen School of Medicine, University of California, Los Angeles, Los Angeles, CA, United States

**Keywords:** chaperone, histone, folding, disease, development, chromatin, nucleosome

## Abstract

Maintenance of protein folding homeostasis, or proteostasis is critical for cell survival as well as for execution of cell type specific biological processes such as muscle cell contractility, neuronal synapse and memory formation, and cell transition from a mitotic to post-mitotic cell type. Cell type specification is driven largely by chromatin organization, which dictates which genes are turned off or on, depending on cell needs and function. Loss of chromatin organization can have catastrophic consequences either on cell survival or cell type specific function. Chromatin organization is highly dependent on organization of nucleosomes, spatiotemporal nucleosome assembly and disassembly, and histone turnover. In this review our goal is to highlight why nucleosome proteostasis is critical for chromatin organization, how this process is mediated by histone chaperones and ATP-dependent chromatin remodelers and outline potential and established mechanisms of disrupted nucleosome proteostasis during disease. Finally, we highlight how these mechanisms of histone turnover and nucleosome proteostasis may conspire with unfolded protein response programs to drive histone turnover in cell growth and development.

## Maintaining nucleosome proteostasis

Across all domains of life, protein quality control is critical for organismal survival. Within organelles such as the mitochondria and endoplasmic reticulum, there is a balance between the proteins that reside within these compartments that give them their function e.g., the electron transport chain proteins that generate ATP in the mitochondria, and the protein folding chaperones that catalyze folding of the organelle’s polypeptides into their final 3D structure, ensuring proper function. During the folding process, chaperones sequester unstable proteins to prevent them from detrimentally interacting with other proteins or other macromolecules, and modulate the kinetics i.e., speed, of folding, and often across several folding cycles, until a stable *and* functional structure is reached (Balchin et al., 2016). Chaperones not only serve to fold newly synthesized proteins and maintain the folding of longer-lived proteins, they also serve to usher “terminally misfolded proteins”, proteins that have gone through several folding cycles without reaching a functional folded state (Balchin et al., 2016), towards dedicated subcellular protein degradation machinery such as the proteasome. Together these chaperone functions stave off accumulation of cytotoxic misfolded protein aggregates; however, when there are more unfolded proteins than there are chaperones to fold them in compartments such as the ER, mitochondria, and cytosol, these compartments increase the activity of their respective unfolded protein responses (UPRs) (Ron and Walter, 2007; Anckar and Sistonen, 2011; Arrieta et al., 2019). Activation of these UPR pathways results in increased expression of the resident protein folding and protein degradation networks. If protein folding homeostasis (also known as proteostasis) is not restored, these same UPRs will then engage in cell death signaling (Doroudgar and Glembotski, 2013). Just as there are dedicated chaperones and protein degradation machinery that co-evolved with the client polypeptides that traverse the ER or power the mitochondria, there is dedicated machinery that has evolved to meet the protein complex assembly and genome folding demands of chromatin (Hammond et al., 2017; Alvarez-Ponce et al., 2019).

The main organizational subunit of chromatin, the nucleosome, is composed of 147 base pairs of DNA wrapped around a protein octamer of two subunits each of histone H2A, H2B, H3 and H4 (Zhou et al., 2019). These histone proteins are extensively post-translationally modified—by some counts, individual cells can have hundreds of distinct modifications on their nucleosomes (Zhao and Garcia, 2015). Several of these modifications have been shown to operate (alone or in combination) to regulate the binding of other proteins, local chromatin accessibility, transcription, DNA repair and other processes. The role of histone modifications in chromatin biology and gene expression is an active area of research that has been reviewed elsewhere (Henikoff and Smith, 2015). Additionally, nucleosome stability is modulated by co-occupancy with linker histones, which influence formation of higher order chromatin structure, as well as by replacement of core histones with variants of histone H2A, H2B, and H3 (Henikoff and Smith, 2015; Hergeth and Schneider, 2015; Martire and Banaszynski, 2020). Maintaining chromatin organization is a formidable task, given that chromatin must participate in process such as genome duplication, mitosis, and cellular differentiation, all while maintaining cell type identity and the ability to respond to physiological and pathophysiological stimuli (Palozola et al., 2019). In this review, our goals are to highlight the specific protein complex assembly challenges associated with maintenance of chromatin, to examine how chromatin function changes during disease and development through the lens of nucleosome and histone turnover, and to shed light on potentially druggable interactions between other protein quality control pathways and the histone chaperone and chromatin remodeling network. The mechanisms by which histone chaperones engage in the folding of histones and nucleosome assembly has been described elsewhere (Hammond et al., 2017).

## The histone chaperone network and ATP-dependent chromatin remodelers in replication-dependent and independent histone turnover

**FIGURE 1:**
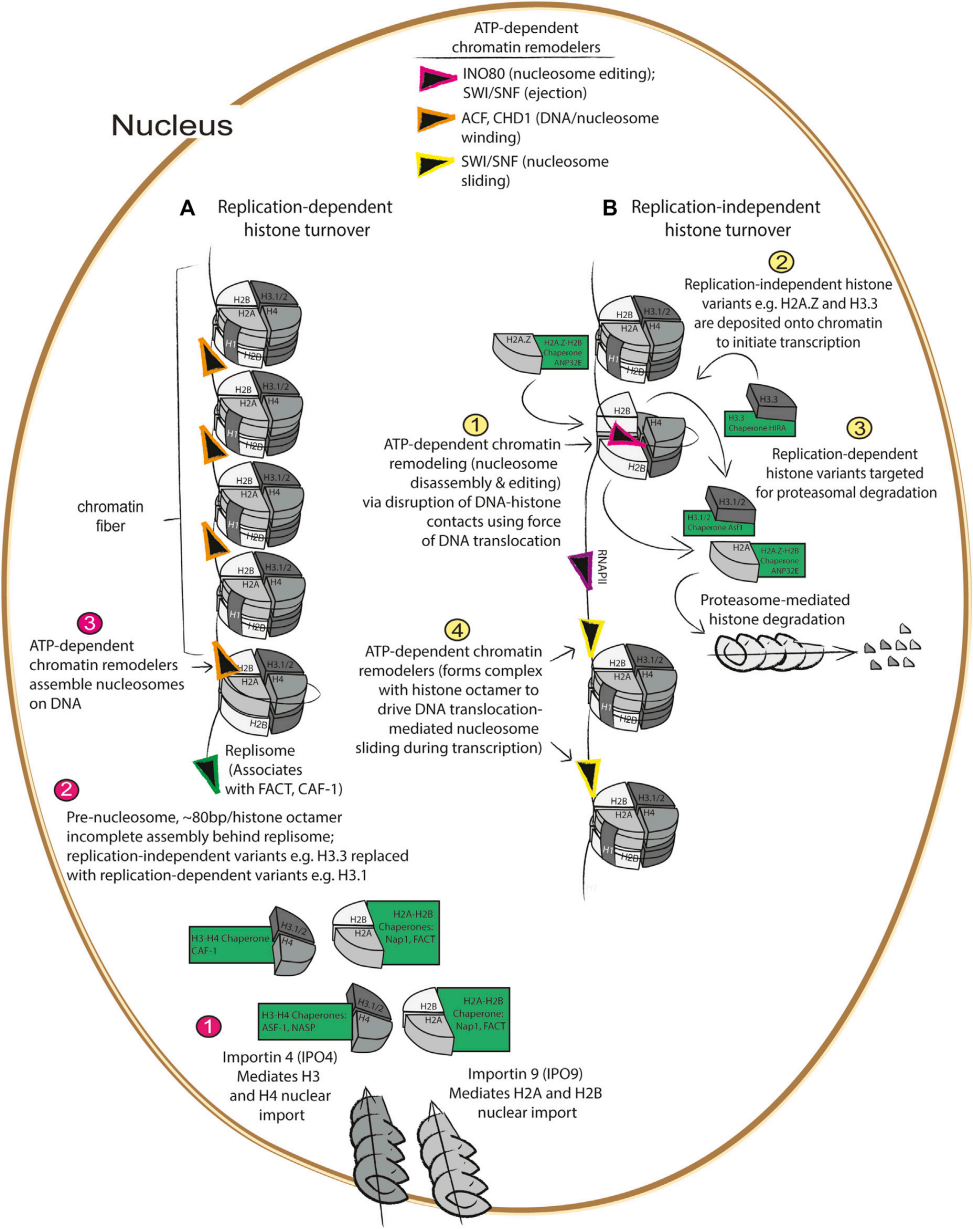
Replication dependent and independent histone turnover mediated by histone chaperones ATP-dependent chromatin remodelers **(A)** 1) Import of newly synthesized histones are imported by importins (Jakel et al., 2002; Soniat et al., 2016) at the nuclear membrane which are then recognized by their cognate histone chaperones where 2) they are assembled with newly synthesized DNA into pre-nucleosomes behind the DNA replisome (Fei et al., 2015), followed by 3) maturation into assembled, higher order nucleosomes via ATP-dependent chromatin remodelers (Lusser et al., 2005; Clapier et al., 2017). **(B)** During gene transcription, 1) nucleosomes are disassembled by histone chaperones and ATP-dependent chromatin remodelers (Konev et al., 2007; Adam et al., 2013; Pchelintsev et al., 2013), 2) replication dependent histones are replaced with replication independent histone variants such as H3.3 and H2A.Z (Alvarez-Ponce et al., 2019), 3) replication-dependent variants are degraded *via* the proteasome (Oh et al., 2020), and 4) ATP-dependent chromatin remodelers mediate nucleosome sliding during transcription (Whitehouse et al., 1999).

The histone chaperone network is the group of chaperones that mediate the various aspects of histone turnover which include histone synthesis, histone deposition onto and ejection from chromatin, histone sequestration and recycling, histone degradation, histone post-translational modification, and nucleosome assembly and disassembly (Hammond et al., 2017). Much like “typical chaperones”, histone chaperones are defined by their ability to shield and sequester histones from forming dysfunctional interactions with other proteins as well as nucleic acids. However, whereas other chaperones are thought to operate at least in part by shielding aggregation-prone hydrophobic protein topologies, histone chaperones must contend with preventing improper electrostatic interactions driven by the net positive charge characteristic of histone proteins (Hammond et al., 2017). Histone chaperones execute these various processes of histone metabolism during DNA replication, termed replication-dependent histone turnover (Figure 1A) and outside of DNA replication, termed replication-independent histone turnover (Figure 1B). Additionally, similar to how other protein quality control processes are ATP-dependent (Stein et al., 2014; Balchin et al., 2016), we will also discuss how the histone chaperone network operates in concert with ATP-dependent chromatin remodelers to mediate various aspects of nucleosome assembly and disassembly during replication-dependent and independent histone turnover.

During replication-dependent histone turnover, newly synthesized histone variants—in conjunction with “old histones” that are thought to contribute to maintenance of epigenetically repressed states (Escobar et al., 2022)—are deposited behind the replisome by histone chaperones onto chromatin during DNA synthesis, resulting in the formation of pre-nucleosomes. Pre-nucleosomes, composed of approximately 80 base pairs loosely wrapped around the histone octamer, are converted into mature nucleosomes *via* DNA translocation by ATP-dependent chromatin remodelers like ACF or CHD1 (Figure 1A) (Lusser et al., 2005; Fei et al., 2015). As DNA is replicated, newly synthesized H3.1/H3.2 and H4 are imported via the nuclear pore localized importin 4 (Soniat et al., 2016) (IPO4), and this histone heterodimer is bound by the histone chaperone anti-silencing factor 1 (Asf1) before being transferred to the chromatin assembly factor (CAF-1) histone chaperone complex that is associated with the DNA replisome (Groth et al., 2005; Groth et al., 2007; Gunesdogan et al., 2014) (Figure 1A). Concurrently, nucleosome assembly protein 1 (Nap1) and/or the facilitates chromatin transaction (FACT) complex mediates nuclear import of H2A/H2B via importin 9 (IPO9) (Jakel et al., 2002), and mediates nucleosome assembly at sites of DNA replication (Aguilar-Gurrieri et al., 2016). While these processes are the essential components of replication-dependent histone turnover, they also interface with various aspects of replication-independent histone turnover (Figure 1B).

Replication-independent histone turnover refers to histone turnover events that occur outside of DNA replication, which include centromere formation, DNA repair, and initiation of transcription (Figure 1B). As part of the cell cycle, centromeres are formed which are critical for mitotic spindle assembly; centromeres are formed when H3 variants are replaced by the centrosome-specific H3 variant, Histone H3-like centromeric protein A (CENPA) which is deposited onto highly repetitive DNA sequences by the CENPA-specific chaperone, Holliday junction recognition protein (HJURP), during the G1 phase of the cell cycle (Dunleavy et al., 2009; Dunleavy et al., 2011). In addition to the role of histone turnover in centromere formation, histone turnover is also a critical aspect of the DNA damage response, as nucleosomes must be disassembled by the histone chaperones and ATP-dependent chromatin remodelers working in concert to allow the DNA to be accessible to the repair machinery (Rogakou et al., 1998). As will be discussed in further detail below, the process of nucleosome disassembly and histone degradation following DNA damage is driven by deposition and phosphorylation of the histone variant H2AX, and this phosphorylated form is referred to as γH2AX (Rogakou et al., 1998; Hauer et al., 2017; Piquet et al., 2018). Deposition of H2AX is mediated by the Facilitator of Chromatin Transactions (FACT) complex, and phosphorylation of H2AX by the kinase known as ataxia-telangiectasia-mutated and Rad3-related (ATR) initiates nucleosome disassembly and recruitment of the DNA damage response machinery, including the ATP-dependent chromatin remodeler and nucleosome editor INO80 [this family of proteins uses DNA translocation to disrupt DNA-histone interaction to mediate histone eviction and replacement (Rogakou et al., 1998; Clapier et al., 2017; Hauer et al., 2017; Piquet et al., 2018)]. Interestingly, H2AX deposition occurs at sites where the histone chaperone ANP32E, together with INO80, mediates eviction of the pro-transcription H2A.Z/H2B heterodimer (Piquet et al., 2018). Following DNA repair, H3.3 is deposited onto newly repaired DNA by the H3.3 histone chaperone histone cell cycle regulator (HIRA) complex, composed of HIRA, ASF-1, and CABIN-1, in conjunction with the ATP-dependent chromatin remodeler CHD1 (Konev et al., 2007) to drive re-initiation of transcription following DNA damage (Adam et al., 2013). These studies are consistent with the notion that deposition of H2A.Z and H3.3 at transcription start sites drives gene transcription. In the following section, we will discuss how various aspects of replication-dependent and independent histone turnover cooperate to ensure fidelity in re-establishment of epigenetic states following mitosis.

## Replication-dependent and independent histone turnover in proliferating cells

**FIGURE 2:**
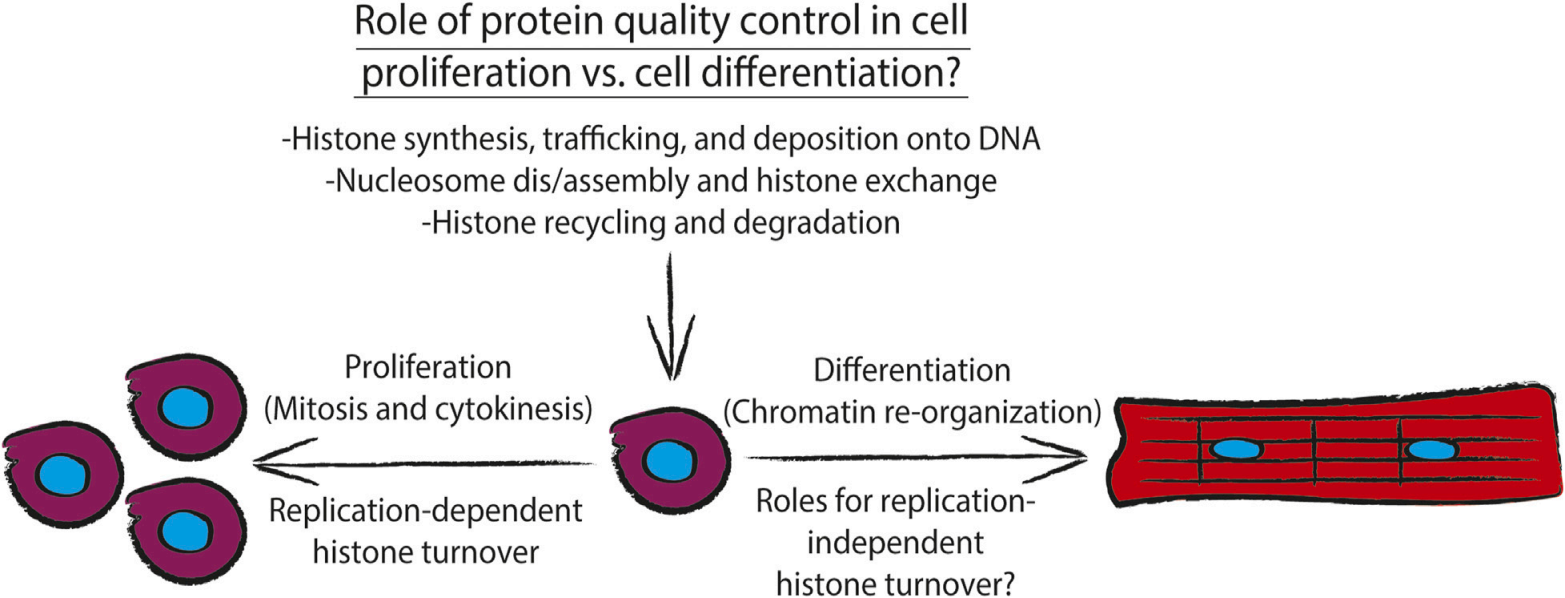
Diagram showing how different aspects of histone metabolism and nucleosome dynamics directly contribute to the cellular task of maintaining protein quality control. During proliferation, DNA replication-dependent histone turnover, *via* concerted histone synthesis, trafficking, and deposition onto chromatin, along with histone recycling and degradation by the histone chaperone network and ATP-dependent chromatin remodelers drive proliferation. Additionally, chromatin must be re-organized to drive cell differentiation. To drive cell differentiation, the histone chaperone network and ATP-dependent chromatin remodelers must execute replication-independent histone turnover which also depends on concerted histone synthesis, trafficking, and deposition onto chromatin, along with histone recycling and degradation of replication-independent histone variants.

During DNA replication (i.e., S phase of the cell cycle), cells must duplicate the entire genome, and as a result must contain approximately twice the number of histone proteins present in a non-dividing cell (Gunesdogan et al., 2014). As discussed above, uncontrolled interactions of histones with DNA or other proteins can be geno- and proteotoxic and can result in genomic instability (Gunjan and Verreault, 2003; Singh et al., 2010). Such catastrophic interactions are avoided during DNA replication by an orchestrated balance between histone synthesis, histone degradation, and histone chaperone activity, which cooperate to maintain nucleosome dynamics (Figure 2). These concerted activities contribute to “epigenetic memory” (Palozola et al., 2019)- the concept that cells are able to remember what gene programs to keep on or off following cell division or other events that force a loss of interphase chromatin territories such as repair of DNA double stranded breaks.

Cellular histone levels are tightly controlled via regulation of their synthesis as well as their degradation during cell proliferation, specifically, during DNA synthesis (Figure 2). It has been demonstrated that in proliferating cultures of the yeast *Saccharomyces cerevisiae*, histone synthesis undergoes sub scaling during cell growth i.e., synthesis of histones is matched to DNA content and phase of the cell cycle, rather than cell size (Marzluff et al., 2008; Swaffer et al., 2021). At the post-transcriptional level, the transcripts encoding replication-dependent histones exhibit a short half-life due to the lack of a stabilizing poly-A tail. However, they are stabilized and trafficked to the translational machinery during S phase by histone stem-loop-binding protein (SLBP), which binds to the 3’ untranslated region of these transcripts and is degraded at the end of S phase in the cell cycle (Zheng et al., 2003).

Following mitosis, during which gene transcription is mostly silent due to a combination of chromatin inaccessibility to transcription machinery and removal of pro-transcriptional histone variants e.g. H2A.Z and H3.3 (Figures 1A,B), the cell must re-initiate gene transcription which is thought to require turnover of replication-dependent histone variants for replication-independent histone variants (discussed further below). Recently, it has been demonstrated in embryonic stem cells that the anaphase promoting complex (APC), which canonically ubiquitylates securin and cyclin B to initiate their proteasome-mediated degradation to drive anaphase (King et al., 1995; Cohen-Fix et al., 1996), also mediates ubiquitylation and proteasome-mediated degradation of histones present at transcription start sites of genes critical to mRNA translation and ribosome function (Oh et al., 2020). This task of extracting histones from chromatin is performed by a AAA-ATPase that is associated with the APC, valosin-containing protein (p97/VCP); VCP-mediated extraction of histones from chromatin is similar to the process of endoplasmic reticulum-associated degradation (ERAD), during which VCP hydrolyzes ATP to extract misfolded ER luminal and transmembrane proteins from the endoplasmic reticulum to allow for their ubiquitylation by the ER transmembrane E3 ubiquitin ligase Hrd1 (Ye et al., 2005). This study (Oh et al., 2020) went on to show that the chromatin associated factor, WDR5, which binds to H3K4 trimethylated histones (H3K4Me3; a mark that is associated with active interphase promoters) recruits the APC along with transcription initiation factors TF-IID and TBP, and the ATP-dependent chromatin remodelers INO80 which mediates nucleosome editing (Figure 1B). These results support the notion that pathways of protein quality control, cell cycle control, and chromatin remodeling cooperate to mediate histone turnover and thus maintenance of cellular identity in dividing cells (Figure 2).

There are several pieces of evidence that members of the histone chaperone network work with ATP-dependent chromatin remodelers to contribute to the maintenance of cellular identity in dividing cells. HIRA, which exists in a complex with chromatin remodelers ISWI, SNF, and Brg1 (Hang et al., 2010; Pchelintsev et al., 2013), is required for the maintenance of cellular identity of C2C12 myoblasts, yet also drives myoblast differentiation into myotubes (Yang et al., 2016; Esteves de Lima et al., 2021). With regard to maintenance of cellular identity in proliferating cells, knockout of HIRA results in loss of satellite muscle stem cell identity as indicated by decreased expression of Pax7, an established marker of satellite muscle stem cells, and of myogenic transcription factor MyoD, along with impaired capacity to differentiate into myotubes. Knockout of *Hira* also increased expression of different lineage markers unrelated to muscle function (e.g., neuronal development and synapse organization) (Esteves de Lima et al., 2021). Consistent with a role for HIRA in driving replication-independent turnover, knockout of *Hira* resulted in decreased levels of H3.3 and H3K27Ac at the promoters of genes encoding skeletal muscle-specific genes. Additionally, there was an increase in H3K4Me3 at gene promoters, along with increased transcript expression, of genes encoding proteins found in other cell and tissue types (Esteves de Lima et al., 2021). The ability of HIRA to engage in depositing and turnover of histone H3.3 is dictated by a phosphorylation switch (Yang et al., 2016). In proliferating C2C12 myoblasts, HIRA was shown to be phosphorylated by Akt1/2, and upon differentiation into myotubes HIRA is dephosphorylated, which was followed by a concomitant increase in expression of myogenic genes e.g. myogenin and myosin heavy chain. Expression of human HIRA containing a non-phosphorylatable S650A point mutation (HIRA_S650A_) was sufficient to drive myoblast differentiation into myotubes and resulted in increased deposition of H3.3 at active gene promoters, as determined by H3.3 ChIP-qPCR for the MyoD promoter. Furthermore, expression of the phosphomimetic mutant, HIRA_S650D_, prevented accumulation of H3.3 at the MyoD promoter. As for how Hira knockout might contribute to increased expression of genes associated with alternative cellular identities, it is possible that loss of this histone chaperone disrupts the Hira complex which, as discussed above, is formed in part by UBN1 and HIRA. Together these two chaperones have been shown to mediate formation of senescence-associated heterochromatin foci i.e. transcriptionally repressed chromatin, which include cell cycle control genes e.g. Cyclin A2 (Banumathy et al., 2009).

Together these studies support the idea that maintenance of epigenetic memory and concomitant chromatin remodeling in proliferating cells is reliant on coordinated activities between post-transcriptional regulation of histone synthesis, protein quality control and cell cycle control pathways, and activities of the histone chaperone network that can be modulated by post-translational modifications (Figure 2).

## Onco-histones and the histone chaperone network

Cancer, as a disease characterized by uncontrolled cell proliferation due in part to gain-of-function mutations in proto-oncogenes and loss-of-function mutations in tumor suppressor genes, heavily relies on robust function of protein quality control networks in order for tumor cells to survive within the tumor microenvironment. For example, inconsistent and low tumor perfusion, due to a mismatch between the rate of tumor growth and tumor-mediated angiogenesis, results in low oxygen and nutrient concentration, thus putting a strain on the energy-dependent aspects of maintaining proteostasis e.g., ATP-mediated protein folding in the endoplasmic reticulum (Tu et al., 2000). In this section, we aim to discuss how mutated histone proteins, known as “onco-histones” (Nacev et al., 2019) drive aggressive tumor proliferation via chromatin remodeling.

**FIGURE 3:**
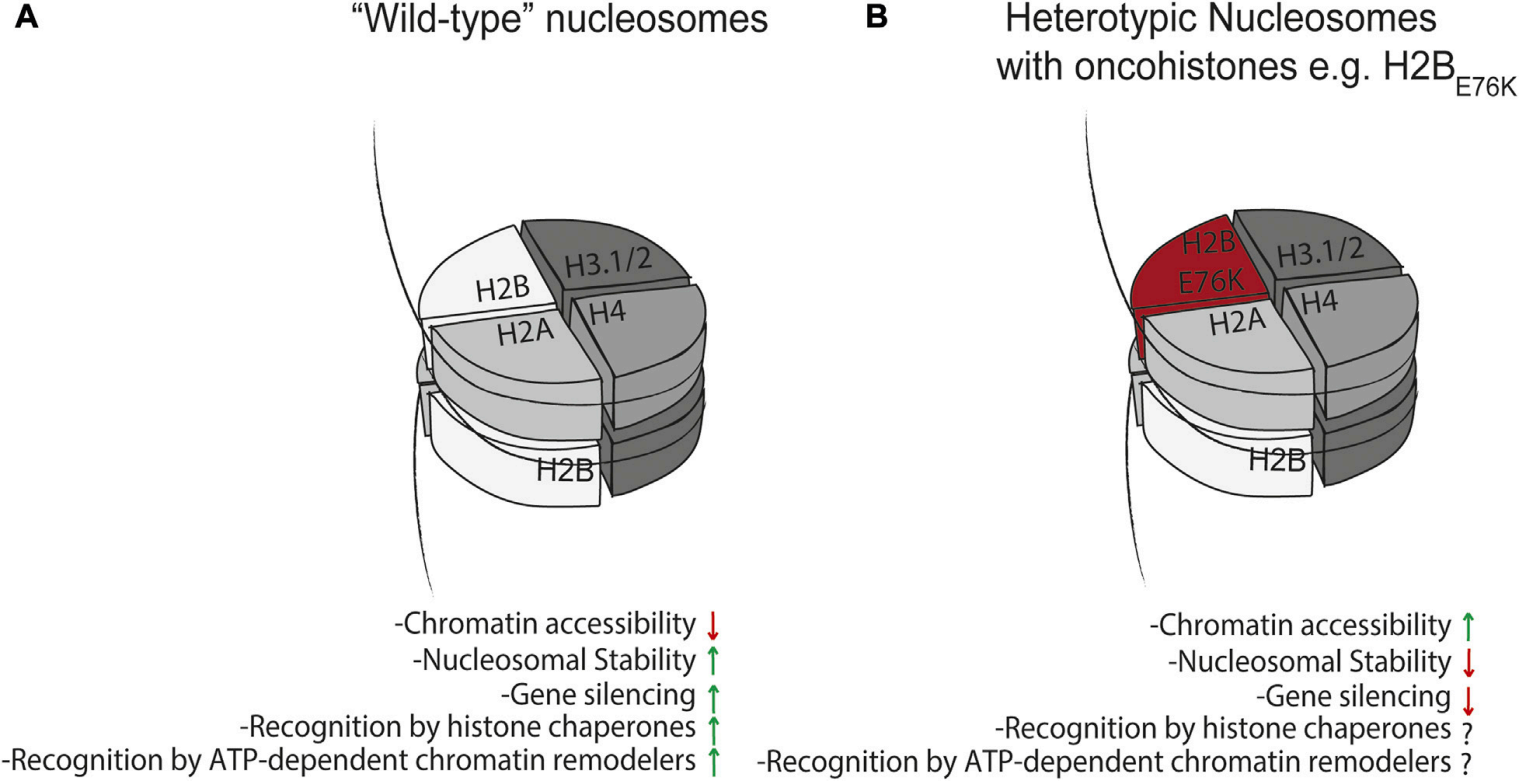
Effect of mutant histones on nucleosome dynamics. **(A)** “Wild-type” nucleosomes can minimize chromatin accessibility, mediate gene silencing, and are recognized as substrates for histone chaperones and ATP-dependent chromatin remodelers. This is in contrast to heterotypic nucleosomes **(B)** containing mutant histones which have been shown to impair nucleosome-mediated gene silencing (Arimura et al., 2018; Bennett et al., 2019; Nacev et al., 2019), and these and other mutant histones may have altered or impaired interactions with the histone chaperone network and ATP-dependent chromatin remodelers. This may in turn affect nucleosome stability and turnover to drive pathology.

There is mounting evidence that cancer cells acquire mutations in histone genes that can result in genome-wide changes in chromatin organization or destabilize interactions of histones within the nucleosome, in turn disrupting gene regulatory mechanisms (Nacev et al., 2019). Mutations can occur within the dimerization interface of histones resulting in nucleosome instability, perhaps resulting in disruption of higher order chromatin structures that are critical in maintenance of cellular identity, or in the histone tails which can alter the affinity post-translational modifiers have for these mutant histones. The poster child of onco-histone mutations is exemplified by a Lys27Met mutation in histone H3.3 (H3.3_K27M_) (Lewis et al., 2013) in pediatric glioblastoma. Additionally, human diffuse intrinsic pontine gliomas (DIPGs) containing this K27M mutation display significantly lower overall amounts of the gene-silencing histone modification H3 lysine 27 trimethylation (H3K27me3) and higher amounts of the gene activating mark H3K27Ac, the former of which the authors demonstrated was due to the H3.3_K27M_ mutant histone directly inhibiting PRC2 methyltransferase activity (Lewis et al., 2013). A critical observation made in this study with the regard to the effects of H3.3_K27M_ on the epigenome is that even though H3.3_K27M_ itself cannot be methylated or acetylated due to the methionine substitution, H3.3_K27M_ only needs to make up a fraction of the total H3 pool (thereby resulting in heterotypic nucleosomes containing a wild-type H3 and mutant H3.3_K27M_) to drive genome wide decreases in H3K27Me3 and increases in H3K27Ac on the remaining wild-type H3 expressed in the cell. In a subsequent study (Nacev et al., 2019) in which onco-histones are defined and catalogued across various tumor contexts, two standout example of how mutations in present in onco-histones are very likely to alter their folding are glycine or proline substitutions at R29 in histone H2A and R39 in histone H4, which is predicted to disrupt the stability of their α-helical folds. These putative onco-histones could theoretically require longer-lived interactions with their cognate chaperones and form highly unstable nucleosomes, although to the best of our knowledge the former has not been formally tested. In support of the argument that mutations in histone proteins result in nucleosome instability, ATAC-seq was used in MCF10A cells expressing wild type histone H2B or H2B_E76K_ (Bennett et al., 2019) to demonstrate that accessible regions in both WT or H2B_E76K_ cells tended to be *more* accessible in the latter. Furthermore, expression of H2B_E76K_ in yeast impaired the ability of cells to execute nucleosome-mediated gene silencing (Figure 3). Additionally, this study demonstrated, via nucleosome thermal stability assay, that nucleosomes containing H2B_E76K_ displayed lower stability. These results, along with a separate study (Arimura et al., 2018) showing the relative instability of nucleosomes containing mutant forms of H3.1, H2A.Z, or H2B, are consistent with the notion that onco-histones do indeed alter the stability of nucleosomes (Figure 3). Whether onco-histones, and the nucleosomes they are incorporated into, are more dependent on the histone chaperone network and ATP-dependent chromatin remodelers to achieve their structure, stability, and incorporation into nucleosomes is unknown (Figure 3), but could be addressed experimentally by directly measuring differential turnover and deposition of onco-histones *in vivo*.

We discussed above the finding that the H3.3_K27M_ onco-histone only needs to make up a fraction of the total H3.3 pool to drive genome-wide decreases in placement of H3K27Me3 (Lewis et al., 2013). This observation suggests that increased concentration of mutation-containing histones via alternative mechanisms like translational infidelity (Kapur and Ackerman, 2018) or somatic cell mutation mosaicism (Frank, 2014) can drive other disease pathologies; like onco-histones, these mutant histones may have altered interactions with the histone chaperone network and ATP-dependent chromatin remodelers for their deposition onto chromatin where they might mediate disease progression via disruption of chromatin organization (Figure 3).

## Histone turnover in the neonatal and post-natal heart

The mammalian heart maintains a certain proliferative myocyte capacity after birth, which is critical to cardiac development and function, that is lost as the organism ages (Porrello et al., 2011; Serpooshan et al., 2017; Bogush et al., 2020). In addition to this proliferative switch, cardiac myocytes undergo a metabolic switch where ATP is generated via glycolysis in the neonatal heart versus fatty acid β-oxidation and oxidative phosphorylation in the adult heart (Lopaschuk et al., 1992). This metabolic switch is thought to be driven by a combination of increased blood oxygen tension, increased circulating fatty acids from the mother’s milk, and increased circulating catecholamine levels (Faxelius et al., 1983; Lopaschuk and Jaswal, 2010), all together enforcing the said switch to oxidative phosphorylation which provides enough ATP to power cardiac contractility. The increased rate of oxidative phosphorylation comes with increased production of reactive oxygen species, which can result in DNA damage and result in myocyte death in cases of cardiac ischemia/reperfusion injury. This series of events provides a unique chromatin remodeling challenge in cardiac myocytes as they must maintain a proliferative capacity after birth, contend with changes in oxygen tension after birth which is thought to mediate mitochondrial ROS generation and ensuing DNA damage, and also engage in transcriptomic reprograming to allow for hypertrophic rather than hyperplastic myocyte growth as the heart develops (Nakada et al., 2017). The first studies to examine histone stoichiometry at the protein level in the diseased heart revealed a decrease in the core to linker ratio during the compensatory hypertrophic growth phase (as measured by H1:H4), concomitant with temporal reprogramming of abundance, and DNA association by, other non-nucleosomal chromatin structural proteins (including HMGs and nucleolin) as the heart transitions from hypertrophy to failure (Franklin et al., 2011; Franklin et al., 2012; Monte et al., 2013; Monte et al., 2016). More recent studies have assessed roles for ATP-dependent chromatin remodelers of the SWI/SNF family in driving maturation of the neonatal heart (Hang et al., 2010), and histone turnover in the adult heart, shedding light on mechanisms that may drive this process.

Since the frequency of adult myocyte proliferation is vanishingly low and inadequate for repair after injury (Eschenhagen et al., 2017), myocytes likely engage primarily in replication-independent histone turnover for maintenance of chromatin structure. To our knowledge, the first study (Li et al., 2019) to examine histone turnover in the heart employed the following approach: Col1a1^tetO−H2B−GFP^ x Rosa26^M2rtTA^ transgenic mice were administered doxycycline-containing water to induce expression of GFP-H2B (i.e., a GFP-H2B pulse), followed by removal of doxycycline-containing water resulting in decreased transgene expression (i.e., an unlabeled H2B chase). Isolated myocytes were then subjected to GFP-H2B ChIP-Seq at various chase times, and the resulting sequencing data was used to calculate rates of genome-wide GFP-H2B turnover. Promoters of genes highly transcribed in cardiac myocytes (i.e., striated-muscle specific gene promoters), and cardiac-specific enhancers containing activating histone marks such as H3K27Ac, demonstrated higher rates of GFP-H2B turnover, as compared to genes with silencing marks or pluripotency enhancers. Two considerations for this study are the use of GFP-tagged H2B and the use of an isoform of H2B (*HIST1H2BJ*) that is normally encoded as a replication-dependent histone variant. With regard to the first point, protein tagging has been shown to alter the stability and/or folding kinetics of the protein it is fused to (Sokolovski et al., 2015), suggesting that turnover of GFP-H2B may not perfectly reflect endogenous H2B turnover due to differences in their stability. With regard to the second point, the replication-dependent and independent forms of H2B rely on different members of the histone chaperone network to mediate their folding, trafficking, and deposition onto chromatin (Hammond et al., 2017) (Figures 1A,B), and thus may reflect different rates of turnover depending on whether they are expressed in a dividing versus a non-dividing cell (Hammond et al., 2017). Interestingly, an orthogonal approach used metabolic labeling with deuterated H_2_O to assess rates of protein turnover in adult hearts treated with isoproterenol (Lam et al., 2014). In this study, only histone H4 showed detectable turnover, although technical considerations with the mass spectrometry-based detection of histones prevents a direct comparison of these studies. Consistent with the notion that cardiac remodeling events drive histone turnover, a recent study employed cardiomyocyte specific Ribo-Seq to identify transcripts engaged with polysomal ribosomes in response to pressure-overload mediated cardiac hypertrophy (Doroudgar et al., 2019). Ribosomes containing a myocyte specific HA-tagged ribosome subunit were purified from transgenic mouse hearts, and ribosome footprints from hypertrophic mouse hearts displayed increased histone H4 as well as histone H2AX transcripts, but no increase in replication-dependent histone variants such as *HIST1H2BJ* and histone H3.1 and H3.2. As will be discussed further below, increased expression of H2AX is consistent with the notion that replication-independent turnover occurs in the adult mammalian heart and is due at least in part to the activities of γH2Ax and the DNA damage response. Knockout of *Hira*, a histone chaperone, in cardiac myocytes resulted in cardiac hypertrophy concomitant with impaired contractility, re-expression of fetal genes, and cardiac fibrosis (Valenzuela et al., 2016). Importantly, the ATP-dependent chromatin remodeler BRG1 which interacts with HIRA, is required for cardiac hypertrophy, and was required for GFP-H2B turnover in the study described above (Hang et al., 2010). Together these studies suggest that replication-independent histone turnover occurs in the adult mammalian heart in response to pathological insult.

As discussed above, the high metabolic capacity of cardiac myocytes i.e., ROS generation as a byproduct of oxidative phosphorylation, suggests that cardiomyocytes constantly engage in a higher level of DNA repair activity, particularly in the days and weeks after birth. If this were the case, activity of the DNA damage response in the neonatal heart would be concomitant with the histone turnover that occurs as a result of DNA damage-mediated nucleosome disassembly, and H3.3/H2A.Z-mediated re-initiation of transcription (Adam et al., 2013; Hauer et al., 2017). This is supported by studies demonstrating that 1) myocyte cell cycle exit and indicators of DNA damage in mice can be reversed with exposure to decreased oxygen tension (Nakada et al., 2017) and 2) that pathological growth of the heart in response to pressure overload is associated with increased expression and nuclear localization of γH2Ax (Nakada et al., 2019), which as described above is an orchestrator of nucleosome disassembly (Rogakou et al., 1998; Hauer et al., 2017; Piquet et al., 2018). Together these studies suggest that ROS is a driver of both cardiac development and disease, and that the histone chaperone network is critical for postnatal growth of cardiac myocytes and maintained function of the heart in the disease state.

Recent studies have demonstrated that the potent ER stress response transcription factor ATF6, which is activated in response to accumulation of misfolded proteins in the ER, is responsible for maintaining protein quality and quantity control mechanisms in other subcellular compartments (Blackwood et al., 2020). Specifically, during ischemia/reperfusion injury ATF6 was shown to transcriptionally upregulate critical members of the oxidative stress response, for example catalase, demonstrating that ATF6 prevents cardiomyocytes from accumulating ROS during ischemia/reperfusion injury (Jin et al., 2017). These results support the notion that perhaps ATF6-mediated induction of catalase can also mitigate oxidative stress in the days after birth where neonates adjust to life with atmospheric versus *in utero* oxygen tension. Additionally, an examination of microarray (Martindale et al., 2006) and RNA-Seq (Blackwood et al., 2019) data sets from mouse hearts expressing the transcriptionally active fragment of ATF6 fused to the mutated estrogen receptor demonstrates a significant upregulation of VCP, which as discussed above is critical for histone turnover in proliferating cells. In considering how and why the ER stress response and the histone chaperone network may conspire to drive histone turnover and therefore development of the heart, it has been recently reported that myocyte binucleation after birth is critical for functional maturation of the heart, and that impairment of this process results in non-compaction cardiomyopathy (Gan et al., 2022). Along with postnatal myocyte proliferation that is critical for development and function of the heart (Serpooshan et al., 2017; Bogush et al., 2020), these bouts of mitotic activity—associated or not with cytokinesis—are likely to require histone turnover and re-initiation of transcription following mitosis or DNA damage, in order to maintain myocyte epigenetic memory following mitosis. Given that mitosis and binucleation have been observed in postnatal rat cardiomyocytes (Li et al., 1997), there is the possibility that the replication- and APC/VCP complex-dependent mechanisms of histone turnover described above (Oh et al., 2020) are at play in the postnatal heart. Consistent with ATP-dependent chromatin remodeler activity (i.e. nucleosome remodeling) in response to injury and cardiac development, the SWI/SNF ATP-dependent nucleosome remodeler BRG1 has been shown to be critical for neonatal cardiac development and pathological cardiac hypertrophy (Hang et al., 2010). Together these observations support the notion, which will ultimately need to be supported with future studies, that upon physiological reperfusion that occurs with mammalian birth, ATF6 transcriptionally induces ER targeted chaperones to protect against ER protein misfolding during myocyte growth, induces catalase to protect against excessive ROS-mediated DNA damage after birth (Figure 2), and induces VCP to mediate re-initiation of genes critical to hypertrophic myocyte growth following myocyte mitosis.

## Conclusions and future directions

In summary, histone quality control and the histone chaperone network and ATP-dependent chromatin remodelers that mediates this process are drivers of organism development and disease, representing potential targets in the treatment of pathologies such as cardiovascular disease and cancer. However, some level of caution must be levied in considering these approaches as much remains to be learned about how, over what time course, and for what cellular function histones are turned over by their cognate chaperones (Figure 2). What is the threshold of what is recognized as a damaged or misfolded histone, and does it change in the context of disease or aging? And do misfolded or alternatively folded histones represent as yet an unexplored “epigenetic mark” that signals for chromatin remodeling by histone chaperones and downstream gene expression? As much as it is important to answer these questions in the context of disease, there is much to be gleaned by returning to the basic science of understanding what drives histone turnover at the molecular level.
